# A retrospective evaluation of patients switched from buprenorphine (subutex) to the buprenorphine/naloxone combination (suboxone)

**DOI:** 10.1186/1747-597X-3-16

**Published:** 2008-06-17

**Authors:** Kaarlo Simojoki, Helena Vorma, Hannu Alho

**Affiliations:** 1 Espoo Treatment and Rehabilitation Center, A-clinic Foundation, Espoo, Finland; 2 Department of Psychiatry, Helsinki University Central Hospital, Helsinki, Finland; 3 Department of Mental Health and Alcohol Research, National Public Health Institute, Helsinki, Finland; 4 Unit of Substance Abuse Medicine, University of Helsinki, Helsinki, Finland

## Abstract

**Background:**

In Finland, buprenorphine (Subutex) is the most abused opioid. In order to curb this problem, many treatment centres transferred ("forced transfer") their buprenorphine patients to the buprenorphine plus naloxone (Suboxone) combination product in late 2003.

**Methods:**

Data from a retrospective study involving five different treatment centers, examining the effects of switching patients to Suboxone, were gathered from 64 opioid-dependent patients who had undergone the medication transfer.

**Results:**

Most patients (90.6%) switched to Suboxone at the same dose of buprenorphine that they had been receiving as Subutex (average 22 mg). The majority of these patients (71.9%) were maintained at the same dose of Suboxone throughout the 4-week study period. During the first 4 weeks, 50% of the patients reported adverse events and at the four month time point, 26.6% reported adverse events. However, due to adverse events one patient only discontinued treatment with Suboxone during the 4-week study period, and five during the four month follow-up period. Of the 26 patients in the follow-up period, Suboxone was misused intravenously once each by 4 patients and twice by 1 patient. These 5 patients all reported that injecting Suboxone was like injecting "nothing" with any euphoria, or that it was a bad experience.

**Conclusion:**

We conclude that when patients are transferred from high doses (> 22 mg) of buprenorphine to the combination product, dose adjustments may be necessary especially in the later phase of the treatment. We recommend that a transfer from Subutex to Suboxone should be carefully discussed and planned in advance with the patients and after the transfer adverse events should be regularly monitored. With regard of buprenorphine IV abuse, the combination product seems to have a less abuse potential than buprenorphine alone.

## Background

Buprenorphine is a mu-opioid receptor partial agonist and kappa-opioid receptor antagonist. The high dose sublingual tablet formulation (Subutex) has proven an effective treatment for opioid dependence [1] and is in use in over 40 countries worldwide. The buprenorphine plus naloxone (Suboxone) combination tablet was developed with the objective of having the same sublingual effectiveness and safety profile as buprenorphine alone (Subutex) but with a lower intravenous (IV) misuse potential [2]. Naloxone, when taken sublingually, is poorly absorbed and should have little or no pharmacological effects [3]. When Suboxone is injected intravenously, naloxone is intended to precipitate withdrawal effects in opioid-dependent users, to attenuate feelings of "drug liking", and to provide a generally unpleasant experience [3]. Suboxone is presently approved for use in the United States, Canada, Australia, New Zealand, Malaysia, and recently in the European Union.

In Finland, the most misused IV opioid during the last few years has been buprenorphine [4] mainly originating from other countries, notably France. As a consequence of abuse, access to and monitoring of the treatment has been very strictly controlled, thus necessitating significant resources. At the initiation of this study approximately 200 patients were receiving treatment with Subutex in Finland. When Suboxone became available under special license in Finland towards the end of 2003, several treatment centers switched their patients rapidly from Subutex to Suboxone as a strategy to curb misuse of buprenorphine.

Preliminary data are available from an open label study involving the switching of patients from low-dose Subutex to Suboxone in Australia indicating a need for a dose increase [5]. In contrast, the relative bioavailability of different buprenorphine formulations under chronic dosing conditions has also been studied, and it is possible that the buprenorphine in Suboxone has a slightly higher sublingual bioavailability than the buprenorphine in Subutex [6]. However, detailed effects of switching therapy from Subutex to Suboxone have not been fully investigated.

The study objectives were threefold: i) to examine the potential effect of a switch on the total dose of buprenorphine, ii) to follow whether the switch to Suboxone deters IV misuse, and iii) to follow whether switching from Subutex to Suboxone results in withdrawal symptoms or other significant adverse reactions.

## Methods

The study was retrospective, involving data collection from 64 opioid-dependent patients who had undergone a switch from Subutex to Suboxone. The inclusion criteria were as follows: opioid dependency (ICD-10); patient stabilized on buprenorphine treatment for at least a month; a buprenorphine dose of at least 12 mg/day, the average buprenorphine daily dose in Finland in 2004 was 21 mg, (however, due to Finnish clinical practice for pregnant or lactating women the maintaining dose is generally lower, ≤ 12 mg); patient switched or attempted to switch from Subutex to Suboxone, and older than 18 years of age. Data were collected from five treatment sites in Finland using structured data collection forms. The five treatment centers were randomly chosen from 20 treatment centers that were using Subutex and had more than 10 patients in buprenorphine treatment. All buprenorphine patients, except four patients with low doses of buprenorphine (pregnant women), in these centers were included in the study. Data were collected for three days during the week of the medication switch and at 1, 2, 3, and 4 weeks following the transfer. The data collection was repeated four months post transfer (follow-up period). The data gathered from patient records prior to the switch (Subutex dose, drug misuse and frequency of clinic visits) is considered as baseline data.

### Ethical Conduct of Study

The study was coordinated by the Department of Mental Health and Alcohol Research of the National Public Health Institute in Finland whose Ethical Committee approved the study protocol. Patient data were collected by the treating physician in each treatment site. Data protection was ensured throughout in accordance with the regulations of the National Public Health Institute. The National Public Health Institute funded the study.

### Treatment Records

The treating physicians at these selected clinics collected data about eligible patients who had undergone a "forced transfer" from Subutex to Suboxone. Data were collected from patient archives of the respective study sites and stored with codes to protect the personal details of the study patients. Physicians recorded the Subutex dose at the day of the transfer, and the Suboxone doses used during the four weeks following the switch. Data four months after the study period were also collected.

Drug misuse measures were recorded as follows: (1) Drug misuse history at the time of entering the clinic; (2) any relevant medical history; (3) signs of buprenorphine misuse before the switch from Subutex to Suboxone and at weeks 1–4 following the switch (as evidenced by the presence of fresh needle marks or patients volunteering the information); (4) signs of misuse of other opioids (urine tests and patient reports); and (5) current misuse of any non-opioid drugs (urine tests and patient reports). Length of non-opioid drug use was also recorded.

Adverse events considered being associated with the switch from Subutex to Suboxone on the day of the switch, days 2 and 3, and weeks 1 to 4 following the switch were recorded. Adverse events four months after the study period were also collected. The MedDRA coding was followed.

Overall patient complacence (patient satisfaction with the switch to Suboxone: yes, no, why not) and compliance, with reasons for non-compliance (where available), were recorded on each of the weekly visits to the clinic before and after the switch. The Finish maintenances treatment legislation (on the time of this study) states that only good patient compliance allows take-home medication, and at the most for 8 days. Thus, the frequency of weekly visits gives an indirect implication of compliance; the less visits the better compliance.

### Study sites

The 5 treatment centers were located in different parts of Finland. Two of the centers are located in Helsinki (HDL, HUS), one in the district of Southern Uusimaa (Espoon A-klinikka), one in central Finland (Tampere), and one in northern Finland (Raahe). All study doctors were either psychiatrists or general practitioners with long experience in maintenance treatment, and had subspecialty in addiction medicine.

### Primary outcomes

Primary outcomes were the dose of buprenorphine before and after switch to Suboxone, and physical signs or patient reports of intravenous misuse of buprenorphine.

### Secondary outcomes

Physical signs of heroin and other opioid abuse/misuse or evident from urine tests, patient's satisfaction/dissatisfaction with Suboxone, and frequency of patient's visits to their treatment clinic (before and after the switch to Suboxone) were recorded.

## Results

### Subject disposition

The records from a total of 64 patients were examined for this study. The mean age and other background demographics are shown in Table 1. By the end of the 4-week study period, one patient discontinued the maintenance treatment program and three patients were transferred back to Subutex. Out of these three patients, one was transferred back to Subutex due to adverse events and the other 2 patient for a lack of compliance on Suboxone. 60 patients (93.8%) continued treatment with Suboxone into the follow-up period.

**Table 1:** Background characteristics of the patients (n = 64)

Gender, male (n)	52
Age, mean (± SD)	29.9 ± 7
Duration of heroin use (month) mean ± SD^a^	72.2 ± 94.2
Buprenorphine treatment (days) mean ± SD	63.3 ± 363
Subutex mean daily treatment dose (mg) ± SD	22.9 ± 5.4

^a^59 patients reported history of heroin use

During the four months follow-up period, the treatment was discontinued with seven (11.7%) patients, five (8.3%) were moved to other treatment sites (no treatment records available), nine (15%) patients were switched back to Subutex, 13 (21.7%) patients were transferred to methadone, and 26 (43.3%) continued with Suboxone. The main reasons for change of treatment or discontinuation were IV misuse of buprenorphine (10 patients), misuse of other drugs (8 patients), adverse events (6 patients) and dissatisfaction with Suboxone (5 patients).

### Buprenorphine dose during the transfer and study periods

#### Weeks 1–4

Fifty-eight patients (90.6%) switched to Suboxone at the same dose of buprenorphine that they had been receiving as Subutex. One patient was transferred with a higher Suboxone dose (2 mg), two with lower doses (2–4 mg) and three patients were "titrated" with daily increases of Suboxone (patient anxiety over the transfer) up to the previous Subutex dose (Table 2).

**Table 2:** Induction and transfer dose (n = 64)

	n (%)
Switched to same dose of Suboxone as Subutex	58 (90.6)
Switched to higher dose of Suboxone than Subutex	1 (1.6)
Switched to lower dose of Suboxone than Subutex	2 (3.1)
Titrated with Subutex during switch	3 (4.7)

^a^59 patients reported history of heroin use

Out of the 60 patients finishing the 4-week study period, 53 patients (82.8%) were treated with Suboxone only throughout the study period, and 46 of these (71.9%) were maintained at the same dose of Suboxone (Table 3). During the 4 week period, 4 patients (6.3%) had dose reductions and 1 patient (1.6%) had a dose increase with Suboxone. Two patients (3.1%) had temporary dose changes during the 4-week study period and seven patients had titrated dosing (2–3 day interval in increase).

**Table 3:** Maintenance dose weeks 1–4 (n = 64)

	n (%)
Treated with Suboxone only throughout 4-week study period	53 (82.8)
Stayed at same dose of Suboxone only throughout 4-week study period	46 (71.9)
Dose reduction	4 (6.3)
Dose increase	1 (1.1)
Temporary dose change	2 (3.1)
Titrated^1 ^with Subutex throughout 4-week study period	7 (11)
Discontinued treatment with Suboxone during 4-week study period	4 (6.2)

^1^patients had at the same time Suboxone and Subutex, first day 50:50 and during the first week gradually to Suboxone only

#### Four month follow-up

Out of the 26 patients continuing treatment with Suboxone, 10 patients (38.5%) were treated with the same dose of Suboxone, 15 patients (57.7%) had dose reductions, and 1 patient (3,8%) had a dose increase

### Drug abuse

At baseline, 9 patients (14.1%) showed physical evidence of IV drug misuse and 7 reported it being buprenorphine and two heroin. Forty-seven patients (73.4%) indicated that they were not abusing buprenorphine or heroin and showed no signs of IV misuse. Information on IV misuse of buprenorphine was not recorded for 8 patients (12.5%). Twenty patients (31.3%) reported abuse of other drugs also (cannabis, amphetamine, benzodiazepines).

At weeks one, two and three, seven, three and six patients, respectively, showed signs of IV abuse of buprenorphine (Subutex or Suboxone). Three patients who had records of IV-misuse at baseline did not continue misusing during the 4-week follow up period, and three new patients with IV-misuse were observed during this period. Over the 4-week study period, there was no evidence of misuse of other opioids.

During the follow-up period the total number of incidents of IV opiate abuse remained at the same, 10 patients were recorded as showing signs of intravenous buprenorphine (Subutex or Suboxone) misuse and 1 patient informed the investigator that he had used heroin. One patient tested positive for codeine, which was permitted for a known complaint. Of the buprenorphine IV abuse, Suboxone was misused intravenously once each by 4 patients and twice by 1 patient. These 5 patients all reported that injecting Suboxone was like injecting "nothing" with any euphoria or that it was a bad experience. Four of them reported also that while they tried to inject Suboxone, they would not repeat the experience.

### Complacence and Compliance

Overall, more than 60% of patients were compliant with Suboxone treatment throughout the 4-week study period. The highest rate of compliance occurred at Centre Raahe where all patients used Suboxone treatment throughout the 4-week study period. During the 4-week observation period, approximately half the patients were satisfied with Suboxone treatment. At centre HDL, patient satisfaction was 28.6% at week 4 while it ranged from 50.0% to 66.7% at the other 4 centers. Clinic visits across the centers varied from once per week to five visits per week. The switch from Subutex to Suboxone did not change the frequency of visits, and frequency did not change over the 4-week study period. The patient satisfaction did not correlate to the number of visits/clinic or the dose of Suboxone used. Twenty-four patients (37.5%) were satisfied with the switch from Subutex to Suboxone every time the question was asked during the 4-week study period, and 26 patients (40.6%) were dissatisfied every time they were asked. The 14 other patients (21.9%) were satisfied at some point during the 4-week study period, and 9 of these patients were satisfied at the end of the 4-week study period. Twenty-six of the 27 patients (96.3%) who were still being treated with Suboxone at the end of the 4-month follow-up were satisfied.

### Adverse events

During the four week study period, 32 of the 64 patients (50.0%) reported adverse events (Table 4). Gastrointestinal adverse events were the most commonly reported adverse events. Nausea and gastrointestinal pain were the most common adverse events, being experienced by 13 (20.3%) and 10 (15.6%) patients, respectively. Other common adverse events were fatigue (8 patients, 12.5%), headache (8 patients, 12.5%), hyperhidrosis (8 patients, 12.5%), vomiting (7 patients, 10.9%), and dyspepsia (7 patients, 10.9%). During the 4-month follow-up period, 16 patients (26.6%) out of the 60 patients who started the follow-up period with Suboxone reported an adverse event (Table 4). One patient discontinued treatment with Suboxone during the 4-week study period due to adverse events, and 5 patients discontinued due to adverse events during the follow-up period. There were no deaths or other serious adverse events reported for patients in the study. There was no apparent relationship between the average Suboxone daily dose taken during the 4-week study period and the reporting of adverse events.

**Table 4:** Summary of adverse events by type and study period.

SOC Preferred Term	Adverse Events 4-Weeks (n)	(%)a	Adverse Events Follow up (n)	(%)b
Patients	64		60	
Patients who Experienced Adverse Events	32	50.0	16	26.6
Palpitations	4	6.3	0	0
Abdominal Pain Upper	3	4.7	3	4.9
Diarrhea	4	6.3	2	3.3
Dyspepsia	7	10.9	0	0
Gastrointestinal Pain	10	15.6	3	4.9
Nausea	13	20.3	8	13.1
Vomiting	7	10.9	2	3.3
Asthenia	2	3.1	2	3.3
Fatigue	8	12.5	9	14.8
Pain	4	6.3	0	0
Headache	8	12.5	6	9.8
Tremor	4	6.3	1	1.6
Insomnia	4	6.3	2	3.3
Irritability	1	1.6	3	4.9
Hyperhidrosis	8	12.5	3	4.9
Rash	2	3.1	2	3.3

a: Percentage based on all 64 patients. b: Percentage based on the 60 patients who were treated with Suboxone during the follow-up period.

## Discussion

The study has several limitations. Due to the retrospective nature of the study, there were no control groups and the results are only descriptive. However, the patient data were used to assess any general trends associated with the switch to Suboxone, which may provide an insight into the best clinical practices for using Suboxone as a replacement for Subutex. Also the number of the patents is relatively low but represents more than 30% of all buprenorphine treated patients in Finland at the study time and nearly all of the patients at the 5 centers. A survival analysis of the client characteristics and history regarding the time to drop out could have been interesting. Given the relatively small sample size and the scope of this study, however, this sample may not have sufficient power to detect any but the strongest patterns. Satisfaction and compliance differed significantly amongst the treatment centers, thus site specific issues might account for findings also.

The main aim of this study was to follow the medication dose and adverse events during a transfer from buprenorphine to buprenorphine-naloxone combination. The possible dose adjustments were decided by the doctors and were based on each individual's weaning, withdrawal symptoms and adverse events.

During the first four weeks, 50% of the patients reported adverse events. However, only one patient discontinued Suboxone due to adverse events or dissatisfaction, and one patient left the treatment program (retention rate of 98.5%). This indicates that the side effects at this point probably might not predict patients' likelihood of staying in treatment. Indeed, patient records indicate that many of the reported adverse events were interpreted as being the result of anxiety about being forced to switch to Suboxone. However, it is notable that during 4 month follow up period only 43.3% of the patients continued with Suboxone and 16 (61.5%) of these 26 patients also reported adverse events. It should be noted anxiety interpretation is more of a speculation than measured assessment.

Interestingly, over half of the patients still on Suboxone during the 4 month follow up asked for a dose reduction of Suboxone. This may indicate that the adverse events could be related to higher buprenorphine serum levels, because buprenorphine in Suboxone has slightly higher sublingual bioavailability than the buprenorphine in Subutex [6]. The dose reductions of Suboxone were mainly done during the follow-up period which may indicate that bioavailability of high dose of Suboxone in the long term should be more thoroughly investigated. It should be noted, however, that the earlier Australian study [5] had found a need for a dose increase, rather than a decrease, when switching from buprenorphine to the combination medication, that could be related to the "low" (12 mg) average dose of Suboxone in that study.

During the follow-up period relative high number of patients (56.7%) discontinued with Suboxone. Most of them were either transferred to methadone (21.7%), back to Subutex (15%), discontinued (11.7%) the treatment or moved to other treatment centers (8.3%, no records). The most common reason for methadone transfer generally in Finland is polydrug abuse. However, in this study misuse of buprenorphine and/or lack of compliance accounted almost half of the discontinuations during the follow-up period. It is possible that psychiatric distress, when patients did not feel confident with the forced transfer from Subutex to Suboxone, may lead to "illegally top-up" with Subutex, that in turn could lead to medication change to methadone. It is possible also, that because the high illicit use of buprenorphine in Finland [5], the treating personnel had a mistrust on patients reports of Suboxone adverse events, and instead of chancing back to Subutex patients were changed to methadone. Thus, the "forced" transfer event could be a unique situation in Finland and may not be present elsewhere i.e. in USA and Canada, where Suboxone is the main medication.

Switching from Subutex to Suboxone did not increase abuse of other opioids. Based on the retrospective nature and small numbers in the study, no solid conclusions about Suboxone diversion can be done. However, interestingly during the follow-up period of the 60 opioid dependent patients, only 5 patients attempted to misuse Suboxone. Furthermore, they reported that while they tried to inject Suboxone, they would not repeat the experience, suggesting that the transfer to Suboxone may also serve as part of an overall strategy to curb misuse of buprenorphine.

In conclusion, a transfer from Subutex to Suboxone should be carefully discussed and planned in advance with the patients and after the transfer adverse events should be regularly monitored. With regard to buprenorphine abuse, the combination product seems to have a more favorable safety profile than treatment with buprenorphine alone. The results of this study suggest that in high dose Suboxone treatment, dose adjustments should be considered, especially in the later phase of the treatment, if patients report an unusually high number of adverse events. Also based on the clinical data gathered it is recommended that transfers should be discussed and planned in advance with the patients to minimize psychiatric distress and lack of compliance leading to lower retention rates.

## Competing interests

The authors declare that they have no competing interests.

## Authors' contributions

HA planned the study design, KS and HV collected the patient data, HA analyzed the data, KS, HV and HA drafted the manuscript, KS revised and finalized, all authors read and approved the final version.

## References

[B1] Strain EC, Stoller K, Walsh SL, Bigelow GE (2000). Effects of buprenorphine versus buprenorphine/naloxone tablets in non-dependent opioid abusers. Psychopharmacology (Berl).

[B2] Correia CJ, Walsh SL, Bigelow GE, Strain EC (2006). Effects associated with double-blind omission of buprenorphine/naloxone over a 98-h period. Psychopharmacology (Berl).

[B3] Stoller KB, Bigelow GE, Walsh SL, Strain EC (2001). Effects of buprenorphine/naloxone in opioid-dependent humans. Psychopharmacology (Berl).

[B4] Alho H, Sinclair D, Vuori E, Holopainen A (2007). Abuse liability of buprenorphine-naloxone tablets in untreated IV drug users. Drug Alcohol Depend.

[B5] Bell J, Byron G, Gibson A, Morris A (2004). A pilot study of buprenorphine-naloxone combination tablet (Suboxone) in treatment of opioid dependence. Drug Alcohol Rev.

[B6] Strain EC, Moody DE, Stoller KB, Walsh SL, Bigelow GE (2004). Relative bioavailability of different buprenorphine formulations under chronic dosing conditions. Drug Alcohol Depend.

